# Crystal structures of the 2:2 complex of 1,1′-(1,2-phenyl­ene)bis­(3-*m*-tolyl­urea) and tetra­butyl­ammonium chloride or bromide

**DOI:** 10.1107/S2056989017009951

**Published:** 2017-08-08

**Authors:** Chao Huang, Ruyu Wang, Xi Shu, Yu Fan, Yue Qi, Shoujian Li, Chuanqin Xia

**Affiliations:** a College of Chemistry, Sichuan University, Chengdu 610064, People’s Republic of China

**Keywords:** crystal structure, phenyl­ene bis-urea, tetra­butyl­ammonium chloride, tetra­butyl­ammonium bromide, anion binding

## Abstract

The title compounds, tetra­butyl­ammonium chloride–1,1′-(1,2-phenyl­ene)bis­(3-*m*-tolyl­urea) (1/1), C_16_H_36_N^+^·Cl^−^·C_22_H_22_N_4_O_2_ or [(*n*-Bu_4_N^+^·Cl^−^)(C_22_H_22_N_4_O_2_)] (I) and tetra­butyl­ammonium bromide–1,1′-(1,2-phenyl­ene)bis­(3-*m*-tolyl­urea) (1/1), C_16_H_36_N^+^·Br^−^·C_22_H_22_N_4_O_2_ or [(n-Bu_4_N^+^·Br^−^)(C_22_H_22_N_4_O_2_)] (II), both comprise a tetra­butyl­ammonium cation, a halide anion and an *ortho*-phenyl­ene bis-urea mol­ecule. Each halide ion shows four N—H⋯*X* (*X* = Cl or Br) inter­actions with two urea receptor sites of different bis-urea moieties. A crystallographic inversion centre leads to the formation of a 2:2 arrangement of two halide anions and two bis-urea mol­ecules. In the crystals, the dihedral angle between the two urea groups of the bis-urea mol­ecule in (I) [defined by the four N atoms, 165.4 (2)°] is slightly smaller than that in (II) [167.4 (2)°], which is probably due to the smaller ionic radius of chloride compared to bromide.

## Abstract 2

The title compounds both comprise a tetra­butyl­ammonium cation, a halide anion and an *ortho*-phenyl­ene bis-urea mol­ecule. Each halide ion shows four N—H⋯*X* (*X* = Cl or Br) inter­actions with two urea receptor sites of different bis-urea moieties. A crystallographic inversion centre leads to the formation of a 2:2 arrangement of two halide anions and two bis-urea mol­ecules.

## Chemical context   

Hydrogen bonding, π–π inter­actions, anion–π inter­actions, halogen bonds, and anion–macrodipole inter­actions are some of the crucial principal forces that determine structure, self-assembly and recognition in chemical and biological systems (Lehn, 1990 ▸; Jentzsch *et al.*, 2013 ▸). Various urea-based anion receptors of varying complexity and sophistication have been designed and prepared (Amendola *et al.*, 2010 ▸; Wei *et al.*, 2011 ▸; Bregovic *et al.*, 2015 ▸). It has been shown that the efficiency of urea to act as a receptor subunit depends on the presence of two parallel polarized N—H fragments, capable of (i) chelating a spherical anion or (ii) donating two parallel hydrogen bonds to the oxygen atoms of a carboxyl­ate or of an inorganic oxoanion (Custelcean, 2013 ▸). In our ongoing research on N-rich organic ligand design and synthesis (Wang *et al.*, 2015 ▸), we report herein the synthesis of the title *ortho*-phenyl­enedi­amine based methyl substituted neutral organic bis-urea receptor **L** and crystal structures of the 2:2 adducts of **L** with tetra­butyl­ammonium chloride (TBACl) or bromide (TBABr) (I) and (II).
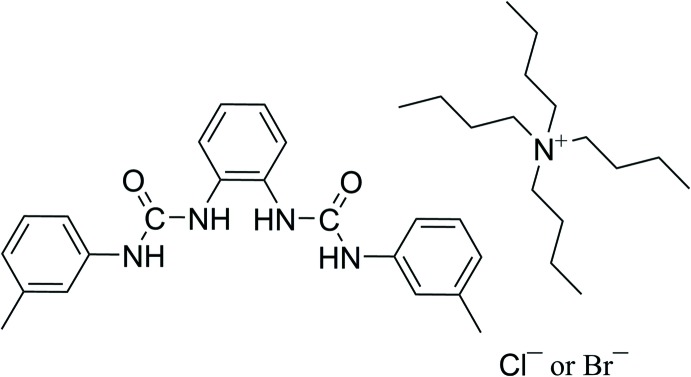


## Structural commentary   

**Figure 1:**
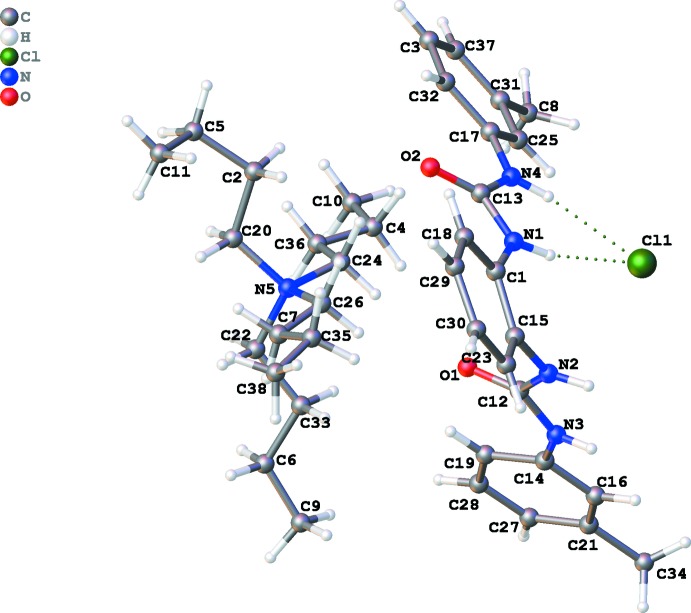
The mol­ecular structure of (I), with atom labels and 50% probability displacement ellipsoids for non-H atoms.

The mol­ecular structures of the title compounds are illustrated in Figs. 1 ▸ and 2 ▸. The receptor **L** displays a *trans* orientation of two urea groups showing non-cooperativity to each other. In the presence of 1.5 equivalents of tetra­butyl­ammonium chloride or bromide in acetone and Et_2_O the 2:2 host–guest complexes (I) and (II) crystallize in the monoclinic space groups *P*2_1_/*n* and *P*2_1_/*c*, respectively. The 2:2 adducts are formed *via* N—H⋯*X* hydrogen bonds between the halide anions and the urea subunits of two bis-urea receptors. Both NH functions of each urea group are *trans* to the C=O double bond across the respective C—N bond, thereby the aromatic substituents are *cis*, with small C_Ar_—N—C=O torsion angles [C1—N1—C13—O2 = 2.7 (4) and C15—N2—C12—O1 = 11.4 (3)° in complex (I), C12—N1—C1—O1 = −0.7 (5) and C14—N3—C13—O2 = 8.5 (4)° in complex (II)]. Moreover, it is also evident that the distance between the two terminal aromatic functions varies considerably due to the torsion angles between the two urea groups and between the two phenyl­ene groups. The angles between the planes through the two urea planes are 55.67 (4)° and 54.51 (5)° in (I) and (II), respectively, with the receptors arranging themselves in a way that in the anion complex the urea groups on the two receptors are oriented in opposite directions therefore establishing inter­actions with two symmetry related anions. This results in the formation of a 2:2 non-capsular assembly *via* non-cooperative equally shared hydrogen-bonding inter­actions between the urea groups and respective anions. This is possibly additionally ascribed for the both *syn* geometrical orientation of the *meta*-substituent (–CH_3_) with respect to the adjacent N—H part of the urea moiety of a particular receptor.

## Supra­molecular features   

**Figure 3:**
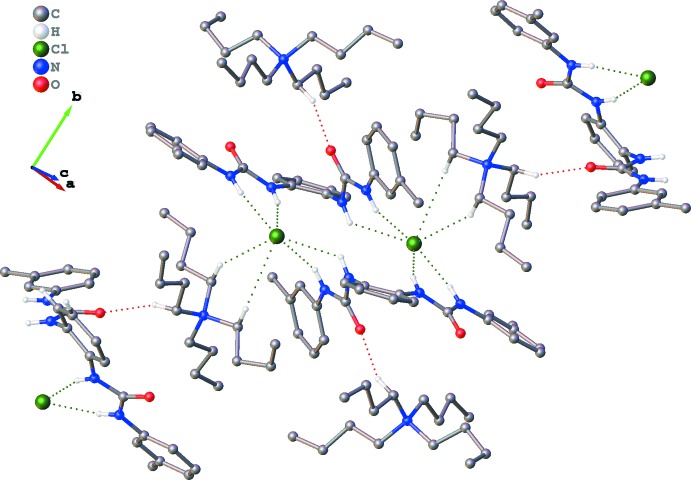
Packing of (I), viewed down the *c* axis, showing one layer of mol­ecules connected by N—H⋯Cl, C—H⋯Cl and C—H⋯O hydrogen bonds (dashed lines). H atoms not involved in hydrogen bonding have been omitted.

Structural elucidation reveals that in complex (I), two symmetry-related chloride anions accept four strong N—H⋯Cl bonds, and similarly two symmetry-related bromide anions accept four strong N—H⋯Br bonds (Tables 1 ▸ and 2 ▸). In addition, the non-capsular assembly of two symmetry-related halide ions and two receptors **L** are additionally stabilized by another two C—H⋯O inter­actions and four weak C—H⋯π supportive inter­actions between the two peripheral TBA units and respective receptor mol­ecules. Additional inter­actions between TBA cations, halide anions and receptor mol­ecules **L** in terms of several short C—H⋯*X* contacts and C—H⋯O contacts connect the 2:2 adducts into infinite layers (Tables 1 ▸ and 2 ▸, Figs. 3 ▸ and 4 ▸). The layers assemble in the 3-D crystal structures (Figs. 3 ▸ and 4 ▸) *via* weak inter­molecular forces. In complex (I), the first inter-layer inter­actions are C22—H22*A*⋯Cl1 and C20—H20*B*⋯Cl1 with C⋯Cl distances of 3.938 (3) and 3.984 (3) Å, respectively; while in complex (II), the C⋯Br distance is 4.003 (3) Å.

## Database survey   

The crystal structure of **L** with a *meta-*substitution of methyl group present in complex (I) and (II) appears not to have been reported previously. However, a search for *ortho*-phenyl­enedi­amine bis-urea with no methyl or any other substitutions on the phenyl ring resulted in some hits. For example, a 1:1 adduct between the bis-urea ligand and benzoate bound in the bis-urea cleft *via* four hydrogen bonds has been reported (Brooks *et al.*, 2005*a* ▸). Similarly, a single terephthalate anion is encapsulated by two bis-urea receptors in another case (Brooks *et al.*, 2005*b* ▸). Furthermore, an *ortho*-phenyl­enedi­amine bis-urea with *para*-nitro substitution receptor has also been reported, three of which enclose one PO_4_^3–^ anion by 12 hydrogen bonds (Li *et al.*, 2013 ▸), whilst the bis-urea isomer with *meta*-nitro substitution displayed good selectivity for carboxyl­ate anions forming a 2:1 complex between receptor and anion (Moore *et al.*, 2013 ▸). Very recently, an *ortho*-phenyl­enedi­amine based 3-chloro-4-methyl disubstituted bis-urea receptor and its isomeric 4-bromo-3-methyl disubstituted bis-urea receptor have been reported and their affinity with the common anions such as Cl^−^, AcO^−^, CO_3_^2–^, SO_4_^2–^ and SiF_6_^2–^ has also been studied (Manna *et al.*, 2016 ▸). Especially, the 4-bromo-3-methyl disubstituted bis-urea forms non-capsular 2:2 host–guest assemblies with chloride ions *via* non-cooperative hydrogen-bonding inter­actions of the urea moieties. This phenomenon is consistent with that of **L** in the present study. Similarly to our work, structural elucidation reveals that two symmetry-identical chloride anions accept four strong N—H⋯Cl bonds [N1⋯Cl 3.226 (6); N2⋯Cl 3.312 (5); N3⋯Cl 3.305 (6); N4⋯Cl 3.270 (6) Å; average 3.278 (8) Å].

## Synthesis and crystallization   

**L**: A solution of 1-iso­cyanato-3-methyl­benzene (0.74 mL, 5.5 mmol) in di­chloro­methane (DCM, 20 mL) was slowly added to a solution of benzene-1,2-di­amine (0.30 g, 2.82 mmol) in DCM (100 mL). The mixture was stirred and refluxed for 24 h. Then the reaction mixture was filtered (glass-filter G4) to give a white precipitate. This precipitate was washed with DCM (40 mL × 3), a mixture solvent of DCM and THF (3:1, 40 mL × 3), and diethyl ether (40 mL × 3), respectively. After that, the solid was collected by filtration. Finally, this solid was dried under vacuum overnight to give **L** as white solid (yield 85%, 0.90 g). m.p: 539–540 K. **MS** (+): *m*/*z* 375.20 [**L**+H]. **^1^H NMR (400 MHz, DMSO-** ***d*** **_6_): δ** 9.01 (*s*, 2H), 8.06 (*s*, 2H), 7.59 (*m*, 2H), 7.32 (*s*, 2H), 7.24 (*d*, *J* = 8.3 Hz, 2H), 7.15 (*t*, *J* = 7.8 Hz, 2H), 7.07 (*m*, 2H), 6.78 (*d*, *J* = 7.4 Hz, 2H), 2.27 (*s*, 6H). **^13^C NMR (400 MHz, DMSO-** ***d*** **_6_): δ** 153.24, 139.81, 137.97, 131.32, 128.67, 124.02, 123.97, 122.57, 118.72, 115.39, 21.29. **FT–IR** (KBr pellet, cm^−1^): 3293, 1636, 1573, 1488, 1297, 1231, 773, 691. Single crystals of complex (I) or (II) suitable for X-ray diffraction were obtained by slow diffusion of an acetone (2 mL) solution of **L** (0.02 mmol) in the presence of TBACl or TBABr (0.03 mmol) in a closed flask with plenty of diethyl ether in three weeks.

## Refinement   

Crystal data, data collection and structure refinement details are summarized in Table 3 ▸. H atoms bonded to N were located from a difference map and refined with distance restraints of N—H = 0.86 (0) Å, and with *U*_iso_(H) = 1.2*U*_eq_(N). Other H atoms were positioned geometrically and refined using a riding model, with C—H = 0.96–0.97 Å and with *U*_iso_(H) = 1.2 (1.5 for methyl groups) times *U*_eq_(C).

## Supplementary Material

Crystal structure: contains datablock(s) I, II, global. DOI: 10.1107/S2056989017009951/im2480sup1.cif

Structure factors: contains datablock(s) I. DOI: 10.1107/S2056989017009951/im2480Isup2.hkl

Supporting information file. DOI: 10.1107/S2056989017009951/im2480Isup4.mol

Structure factors: contains datablock(s) II. DOI: 10.1107/S2056989017009951/im2480IIsup3.hkl

Supporting information file. DOI: 10.1107/S2056989017009951/im2480IIsup5.mol

CCDC references: 1552391, 1556706

Additional supporting information:  crystallographic information; 3D view; checkCIF report

## Figures and Tables

**Figure 2:**
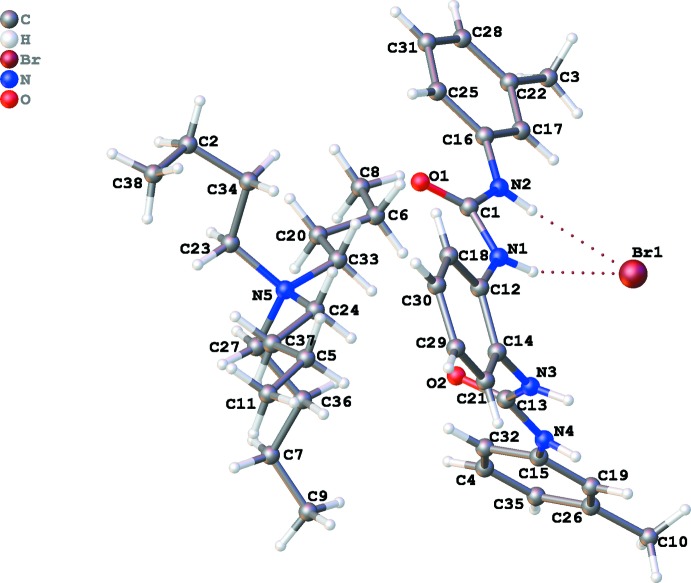
The mol­ecular structure of (II), with atom labels and 50% probability displacement ellipsoids for non-H atoms.

**Figure 4:**
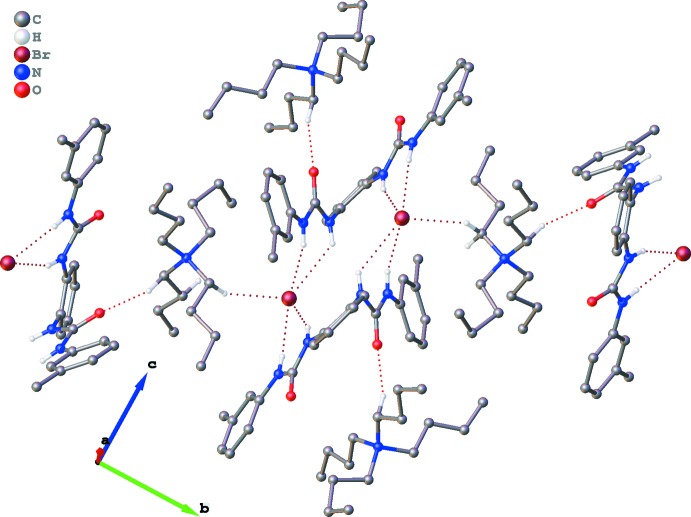
Packing of (II), viewed along the *a* axis, showing one layer of mol­ecules connected by N—H⋯Br, C—H⋯Br and C—H⋯O hydrogen bonds (dashed lines). H atoms not involved in hydrogen bonding have been omitted.

**Table 1:** Hydrogen-bond geometry (Å, °) for (I)

*D*—H⋯*A*	*D*—H	H⋯*A*	*D*⋯*A*	*D*—H⋯*A*
N1—H1⋯Cl1	0.86 (1)	2.53 (1)	3.348 (2)	159 (1)
N2^i^—H2^i^⋯Cl1	0.86 (1)	2.62 (1)	3.231 (2)	129 (1)
N3^i^—H3^i^⋯Cl1	0.86 (1)	2.55 (1)	3.285 (2)	144 (1)
N4—H4⋯Cl1	0.86 (1)	2.34 (1)	3.191 (2)	169 (1)
C26—H26*a*⋯O1	0.97 (1)	2.38 (1)	3.307 (3)	159 (1)
C22^ii^—H22*a* ^ii^⋯Cl1	0.97 (1)	3.05 (1)	3.938 (3)	152 (1)
C20^ii^—H20*b* ^ii^⋯Cl1	0.97 (1)	3.11 (1)	3.984 (3)	150 (1)

Symmetry codes: (i) 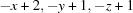; (ii) 
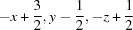
.

**Table 2:** Hydrogen-bond geometry (Å, °) for (II)

*D*—H⋯*A*	*D*—H	H⋯*A*	*D*⋯*A*	*D*—H⋯*A*
N1—H1⋯Br1	0.86 (1)	2.75 (1)	3.557 (2)	157 (1)
N2—H2⋯Br1	0.86 (1)	2.51 (1)	3.359 (2)	168 (1)
N3—H3⋯Br1^i^	0.86 (1)	2.76 (1)	3.420 (2)	135 (1)
N4—H4⋯Br1^i^	0.86 (1)	2.63 (1)	3.417 (2)	152 (1)
C24—H24*a*⋯O2	0.97 (1)	2.38 (1)	3.312 (4)	162 (1)
C27^ii^—H27*a* ^ii^⋯Br1^i^	0.97 (1)	3.10 (1)	4.003 (3)	156 (1)

Symmetry codes: (i) 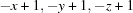; (ii) 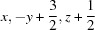.

**Table 3:** Experimental details

	(I)	(II)
Crystal data		
Chemical formula	C_16_H_36_N^+^·Cl^−^·C_22_H_22_N_4_O_2_	C_16_H_36_N^+^·Br^−^·C_22_H_22_N_4_O_2_
*M*_r_	652.34	696.80
Crystal system, space group	Monoclinic, *P*2_1_/*n*	Monoclinic, *P*2_1_/*c*
Temperature (K)	294	294
*a*, *b*, *c* (Å)	13.5654 (4), 20.0993 (6), 14.3329 (4)	10.5879 (2), 20.3165 (5), 18.0828 (3)
β (°)	99.658 (3)	91.0672 (17)
*V* (Å^3^)	3852.53 (19)	3889.11 (14)
*Z*	4	4
Radiation type	Cu *K*α	Cu *K*α
μ (mm^−1^)	1.16	1.72
Crystal size (mm)	0.7 × 0.4 × 0.15	0.5 × 0.3 × 0.2

Data collection		
Diffractometer	Agilent New Gemini, Dual, Cu at zero, EosS2	Agilent New Gemini, Dual, Cu at zero, EosS2
Absorption correction	Multi-scan (*CrysAlis PRO*; Agilent, 2014 ▸)	Multi-scan (*CrysAlis PRO*; Agilent, 2014 ▸)
*T*_min_, *T*_max_	0.608, 1.000	0.444, 1.000
No. of measured, independent and observed [*I* > 2σ(*I*)] reflections	21128, 7530, 5930	21853, 7582, 6081
*R*_int_	0.033	0.039
(sin θ/λ)_max_ (Å^−1^)	0.619	0.618

Refinement		
*R*[*F* ^2^ > 2σ(*F* ^2^)], *wR*(*F* ^2^), *S*	0.081, 0.246, 1.03	0.063, 0.183, 1.03
No. of reflections	7530	7582
No. of parameters	421	421
H-atom treatment	H-atom parameters constrained	H-atom parameters constrained
Δρ_max_, Δρ_min_ (e Å^−3^)	0.83, −0.26	0.95, −0.40

Computer programs: *CrysAlis PRO* (Agilent, 2014 ▸), *SHELXT* (Sheldrick, 2015 ▸), *olex2.refine* (Bourhis *et al.*, 2015 ▸) and *OLEX2* (Dolomanov *et al.*, 2009 ▸).

## supplementary crystallographic information

### Tetrabutylammonium chloride–1,1'-(1,2-phenylene)bis(3-m-tolylurea) (1/1) (I) Crystal data

**Table:**

C_16_H_36_N^+^·Cl^−^·C_22_H_22_N_4_O_2_	*F*(000) = 1421.2282
*M**_r_* = 652.34	*D*_x_ = 1.125 Mg m^−^^3^
Monoclinic, *P*2_1_/*n*	Cu *K*α radiation, λ = 1.54184 Å
*a* = 13.5654 (4) Å	Cell parameters from 6812 reflections
*b* = 20.0993 (6) Å	θ = 4.7–72.2°
*c* = 14.3329 (4) Å	µ = 1.16 mm^−^^1^
β = 99.658 (3)°	*T* = 294 K
*V* = 3852.53 (19) Å^3^	Plate, clear light colourless
*Z* = 4	0.7 × 0.4 × 0.15 mm

### Tetrabutylammonium chloride–1,1'-(1,2-phenylene)bis(3-m-tolylurea) (1/1) (I) Data collection

**Table:**

Agilent New Gemini, Dual, Cu at zero, EosS2 diffractometer	5929 reflections with *I*≥ 2u(*I*)
Detector resolution: 15.9595 pixels mm^-1^	*R*_int_ = 0.033
ω scans	θ_max_ = 72.6°, θ_min_ = 4.4°
Absorption correction: multi-scan (CrysAlis PRO; Agilent, 2014)	*h* = −16→16
*T*_min_ = 0.608, *T*_max_ = 1.000	*k* = −24→24
21128 measured reflections	*l* = −17→11
7530 independent reflections	

### Tetrabutylammonium chloride–1,1'-(1,2-phenylene)bis(3-m-tolylurea) (1/1) (I) Refinement

**Table:**

Refinement on *F*^2^	0 restraints
Least-squares matrix: full	92 constraints
*R*[*F*^2^ > 2σ(*F*^2^)] = 0.082	H-atom parameters constrained
*wR*(*F*^2^) = 0.252	*w* = 1/[σ^2^(*F*_o_^2^) + (0.1696*P*)^2^ + 0.5478*P*] where *P* = (*F*_o_^2^ + 2*F*_c_^2^)/3
*S* = 1.05	(Δ/σ)_max_ = 0.001
7530 reflections	Δρ_max_ = 0.85 e Å^−^^3^
421 parameters	Δρ_min_ = −0.30 e Å^−^^3^

### Tetrabutylammonium chloride–1,1'-(1,2-phenylene)bis(3-m-tolylurea) (1/1) (I) Fractional atomic coordinates and isotropic or equivalent isotropic displacement parameters (Å^2^)

**Table:**

	*x*	*y*	*z*	*U*_iso_*/*U*_eq_	
Cl1	0.81049 (4)	0.43129 (4)	0.44872 (4)	0.0698 (2)	
O1	0.85937 (14)	0.63139 (10)	0.37918 (12)	0.0693 (5)	
O2	0.71380 (15)	0.53977 (10)	0.12843 (14)	0.0753 (5)	
N1	0.83702 (15)	0.51476 (11)	0.25233 (13)	0.0604 (5)	
H1	0.84799 (15)	0.49495 (11)	0.30626 (13)	0.0725 (6)*	
N2	0.99507 (14)	0.56294 (10)	0.38683 (13)	0.0551 (4)	
H2	1.03513 (14)	0.53715 (10)	0.42311 (13)	0.0661 (5)*	
N3	0.94863 (15)	0.60015 (10)	0.52227 (13)	0.0565 (5)	
H3	1.00121 (15)	0.57846 (10)	0.54698 (13)	0.0678 (5)*	
N4	0.67823 (16)	0.48559 (12)	0.25880 (16)	0.0679 (6)	
H4	0.70649 (16)	0.46779 (12)	0.31106 (16)	0.0815 (7)*	
N5	0.65966 (16)	0.73314 (11)	0.19192 (16)	0.0653 (5)	
C1	0.91976 (18)	0.54235 (12)	0.22120 (16)	0.0570 (5)	
C12	0.92846 (16)	0.60121 (11)	0.42575 (15)	0.0518 (5)	
C13	0.74037 (18)	0.51570 (11)	0.20661 (16)	0.0575 (5)	
C14	0.89440 (16)	0.62999 (11)	0.58629 (16)	0.0528 (5)	
C15	0.99990 (18)	0.56459 (11)	0.28853 (16)	0.0553 (5)	
C16	0.91973 (19)	0.61169 (12)	0.68051 (16)	0.0598 (5)	
H16	0.96985 (19)	0.58028 (12)	0.69760 (16)	0.0718 (7)*	
C17	0.57262 (19)	0.47988 (13)	0.2378 (2)	0.0696 (7)	
C18	0.930 (4) (2)	0.54390 (15)	0.12578 (18)	0.0710 (7)	
H18	0.8769 (2)	0.53110 (15)	0.07955 (18)	0.0852 (8)*	
C19	0.82081 (19)	0.67721 (14)	0.5609 (2)	0.0679 (6)	
H19	0.80310 (19)	0.69039 (14)	0.4982 (2)	0.0815 (8)*	
C20	0.5976 (2)	0.74753 (15)	0.0958 (2)	0.0739 (7)	
H20a	0.5332 (2)	0.76464 (15)	0.1054 (2)	0.0887 (9)*	
H20b	0.6305 (2)	0.78241 (15)	0.0658 (2)	0.0887 (9)*	
C21	0.8723 (2)	0.63902 (15)	0.74986 (19)	0.0713 (7)	
C22	0.6638 (2)	0.79804 (13)	0.2472 (2)	0.0711 (7)	
H22a	0.6943 (2)	0.83168 (13)	0.2128 (2)	0.0853 (8)*	
H22b	0.5958 (2)	0.81222 (13)	0.2491 (2)	0.0853 (8)*	
C23	1.0887 (2)	0.58348 (15)	0.2611 (2)	0.0719 (7)	
H23	1.1424 (2)	0.59659 (15)	0.3066 (2)	0.0863 (8)*	
C24	0.6127 (2)	0.67819 (14)	0.2424 (2)	0.0738 (7)	
H24a	0.6110 (2)	0.63794 (14)	0.2048 (2)	0.0886 (8)*	
H24b	0.6556 (2)	0.66962 (14)	0.3025 (2)	0.0886 (8)*	
C25	0.5249 (2)	0.46665 (15)	0.3142 (3)	0.0791 (8)	
H25	0.5632 (2)	0.46068 (15)	0.3738 (3)	0.0950 (10)*	
C26	0.7635 (2)	0.70980 (14)	0.1819 (2)	0.0711 (7)	
H26a	0.7990 (2)	0.69790 (14)	0.2442 (2)	0.0853 (8)*	
H26b	0.7571 (2)	0.66975 (14)	0.1436 (2)	0.0853 (8)*	
C27	0.7982 (2)	0.68588 (18)	0.7230 (2)	0.0834 (8)	
H27	0.7649 (2)	0.70469 (18)	0.7682 (2)	0.1001 (10)*	
C28	0.7738 (2)	0.70463 (17)	0.6306 (3)	0.0843 (8)	
H28	0.7244 (2)	0.73659 (17)	0.6139 (3)	0.1011 (10)*	
C29	1.0185 (3)	0.56429 (18)	0.1000 (2)	0.0852 (9)	
H29	1.0241 (3)	0.56538 (18)	0.0363 (2)	0.1023 (11)*	
C30	1.0984 (2)	0.58304 (19)	0.1665 (2)	0.0844 (9)	
H30	1.1584 (2)	0.59533 (19)	0.1482 (2)	0.1013 (10)*	
C31	0.4218 (2)	0.46212 (17)	0.3040 (3)	0.0952 (11)	
C32	0.5179 (2)	0.48578 (18)	0.1479 (3)	0.0880 (9)	
H32	0.5489 (2)	0.49406 (18)	0.0959 (3)	0.1056 (11)*	
C33	0.7197 (3)	0.79566 (16)	0.3467 (2)	0.0840 (8)	
H33a	0.6880 (3)	0.76364 (16)	0.3828 (2)	0.1008 (10)*	
H33b	0.7876 (3)	0.78073 (16)	0.3460 (2)	0.1008 (10)*	
C34	0.9012 (3)	0.6187 (2)	0.8510 (2)	0.1019 (12)	
H34a	0.919 (3)	0.5725 (5)	0.8541 (2)	0.1528 (18)*	
H34b	0.9571 (17)	0.6450 (12)	0.8802 (7)	0.1528 (18)*	
H34c	0.8458 (9)	0.6256 (16)	0.8836 (6)	0.1528 (18)*	
C35	0.8258 (3)	0.7589 (2)	0.1387 (3)	0.0981 (11)	
H35a	0.7871 (3)	0.7762 (2)	0.0808 (3)	0.1177 (13)*	
H35b	0.8426 (3)	0.7958 (2)	0.1820 (3)	0.1177 (13)*	
C36	0.5078 (3)	0.69210 (19)	0.2612 (3)	0.0952 (10)	
H36a	0.5067 (3)	0.73500 (19)	0.2920 (3)	0.1142 (12)*	
H36b	0.4621 (3)	0.69420 (19)	0.2014 (3)	0.1142 (12)*	
C37	0.3671 (3)	0.4680 (2)	0.2134 (4)	0.1108 (14)	
H37	0.2978 (3)	0.4646 (2)	0.2044 (4)	0.1329 (17)*	
C2	0.5797 (3)	0.6888 (2)	0.0283 (3)	0.0994 (11)	
H2a	0.6427 (3)	0.6669 (2)	0.0248 (3)	0.1193 (13)*	
H2b	0.5360 (3)	0.6569 (2)	0.0514 (3)	0.1193 (13)*	
C3	0.4133 (3)	0.4788 (2)	0.1376 (4)	0.1123 (14)	
H3a	0.3749 (3)	0.4817 (2)	0.0775 (4)	0.1348 (17)*	
C4	0.4732 (4)	0.6402 (2)	0.3216 (4)	0.1255 (17)	
H4a	0.4811 (4)	0.5969 (2)	0.2937 (4)	0.151 (2)*	
H4b	0.5155 (4)	0.6411 (2)	0.3833 (4)	0.151 (2)*	
C5	0.5325 (3)	0.7129 (3)	−0.0692 (3)	0.1108 (13)	
H5a	0.5064 (3)	0.6747 (3)	−0.1069 (3)	0.1330 (16)*	
H5b	0.4764 (3)	0.7414 (3)	−0.0628 (3)	0.1330 (16)*	
C6	0.7226 (4)	0.86171 (19)	0.3941 (3)	0.1203 (16)	
H6a	0.7506 (4)	0.89336 (19)	0.3546 (3)	0.1443 (19)*	
H6b	0.6541 (4)	0.87517 (19)	0.3948 (3)	0.1443 (19)*	
C7	0.9182 (3)	0.7290 (3)	0.1176 (4)	0.1218 (15)	
H7a	0.9528 (3)	0.7077 (3)	0.1746 (4)	0.1462 (18)*	
H7b	0.9004 (3)	0.6944 (3)	0.0705 (4)	0.1462 (18)*	
C8	0.3717 (3)	0.4521 (3)	0.3896 (4)	0.1278 (17)	
H8a	0.4103 (17)	0.4215 (16)	0.4325 (14)	0.192 (3)*	
H8b	0.367 (3)	0.4939 (4)	0.4209 (18)	0.192 (3)*	
H8c	0.3058 (12)	0.434 (2)	0.3699 (5)	0.192 (3)*	
C9	0.7765 (4)	0.8685 (3)	0.4886 (3)	0.1283 (17)	
H9a	0.8434 (12)	0.852 (2)	0.4912 (8)	0.193 (2)*	
H9b	0.779 (3)	0.9145 (4)	0.5067 (13)	0.193 (2)*	
H9c	0.744 (2)	0.8432 (19)	0.5312 (6)	0.193 (2)*	
C10	0.3654 (4)	0.6483 (3)	0.3345 (5)	0.145 (2)	
H10a	0.3229 (5)	0.648 (3)	0.2738 (6)	0.218 (3)*	
H10b	0.3471 (13)	0.6123 (14)	0.372 (3)	0.218 (3)*	
H10c	0.3577 (9)	0.6897 (13)	0.366 (3)	0.218 (3)*	
C11	0.5996 (6)	0.7489 (4)	−0.1193 (4)	0.175 (3)	
H11a	0.568 (2)	0.755 (3)	−0.1838 (13)	0.262 (4)*	
H11b	0.615 (4)	0.7912 (13)	−0.090 (3)	0.262 (4)*	
H11c	0.660 (2)	0.7238 (15)	−0.118 (4)	0.262 (4)*	
C38	0.9877 (4)	0.7749 (4)	0.0829 (5)	0.175 (3)	
H38a	0.9520 (10)	0.802 (2)	0.033 (3)	0.263 (4)*	
H38b	1.018 (4)	0.803 (2)	0.1337 (13)	0.263 (4)*	
H38c	1.039 (3)	0.7500 (4)	0.060 (4)	0.263 (4)*	

### Tetrabutylammonium chloride–1,1'-(1,2-phenylene)bis(3-m-tolylurea) (1/1) (I) Atomic displacement parameters (Å^2^)

**Table:**

	*U*^11^	*U*^22^	*U*^33^	*U*^12^	*U*^13^	*U*^23^
Cl1	0.0563 (4)	0.0869 (5)	0.0615 (4)	−0.0022 (3)	−0.0042 (3)	0.0081 (3)
O1	0.0704 (10)	0.0777 (11)	0.0550 (9)	0.0177 (9)	−0.0033 (8)	0.0082 (8)
O2	0.0770 (12)	0.0736 (11)	0.0672 (11)	0.0037 (9)	−0.0112 (9)	0.0113 (9)
N1	0.0634 (11)	0.0659 (11)	0.0478 (10)	−0.0068 (9)	−0.0022 (8)	0.0049 (8)
N2	0.0512 (9)	0.0626 (11)	0.0482 (9)	0.0017 (8)	−0.0007 (7)	0.0031 (8)
N3	0.0558 (10)	0.0632 (11)	0.0476 (9)	0.0098 (8)	0.0000 (7)	0.0029 (8)
N4	0.0552 (11)	0.0790 (14)	0.0651 (12)	0.0030 (10)	−0.0025 (9)	0.0085 (10)
N5	0.0651 (12)	0.0580 (11)	0.0705 (13)	0.0169 (9)	0.0047 (10)	0.0125 (9)
C1	0.0635 (13)	0.0538 (11)	0.0508 (12)	0.0023 (10)	0.0017 (10)	−0.0001 (9)
C12	0.0519 (11)	0.0517 (11)	0.0489 (11)	−0.0033 (9)	−0.0003 (8)	0.0043 (9)
C13	0.0628 (13)	0.0502 (11)	0.0536 (12)	0.0052 (9)	−0.0074 (10)	−0.0039 (9)
C14	0.0515 (11)	0.0507 (11)	0.0549 (11)	−0.0066 (9)	0.0055 (9)	0.0009 (9)
C15	0.0581 (12)	0.0563 (12)	0.0503 (11)	0.0018 (9)	0.0054 (9)	0.0007 (9)
C16	0.0666 (13)	0.0579 (12)	0.0522 (12)	−0.0044 (10)	0.0016 (10)	−0.0016 (10)
C17	0.0568 (13)	0.0585 (13)	0.0871 (18)	0.0039 (10)	−0.0066 (12)	−0.0026 (12)
C18	0.0850 (18)	0.0746 (16)	0.0507 (13)	0.0014 (14)	0.0035 (12)	−0.0038 (11)
C19	0.0623 (14)	0.0705 (15)	0.0724 (15)	0.0086 (11)	0.0152 (11)	0.0143 (12)
C20	0.0713 (16)	0.0718 (16)	0.0750 (16)	0.0209 (13)	0.0017 (13)	0.0122 (13)
C21	0.0807 (17)	0.0726 (16)	0.0624 (15)	−0.0123 (13)	0.0176 (13)	−0.0069 (12)
C22	0.0760 (16)	0.0559 (13)	0.0793 (17)	0.0106 (12)	0.0073 (13)	0.0100 (12)
C23	0.0634 (14)	0.0817 (17)	0.0717 (16)	−0.0078 (13)	0.0147 (12)	−0.0012 (13)
C24	0.0809 (17)	0.0563 (14)	0.0828 (18)	0.0094 (12)	0.0100 (14)	0.0092 (12)
C25	0.0634 (15)	0.0672 (16)	0.105 (2)	−0.0072 (12)	0.0078 (15)	0.0023 (15)
C26	0.0642 (14)	0.0692 (15)	0.0769 (16)	0.0211 (12)	0.0033 (12)	0.0134 (13)
C27	0.0841 (19)	0.087 (2)	0.086 (2)	0.0009 (16)	0.0334 (16)	−0.0125 (16)
C28	0.0747 (17)	0.0857 (19)	0.098 (2)	0.0188 (15)	0.0294 (16)	0.0091 (17)
C29	0.107 (2)	0.096 (2)	0.0573 (15)	0.0050 (17)	0.0273 (16)	0.0016 (14)
C30	0.0769 (18)	0.106 (2)	0.0759 (18)	−0.0048 (16)	0.0277 (15)	0.0040 (16)
C31	0.0665 (17)	0.0706 (18)	0.148 (3)	−0.0114 (14)	0.016 (2)	0.0047 (19)
C32	0.0650 (16)	0.093 (2)	0.095 (2)	0.0048 (15)	−0.0167 (15)	−0.0013 (17)
C33	0.100 (2)	0.0681 (16)	0.0803 (19)	0.0143 (15)	0.0057 (16)	−0.0003 (14)
C34	0.124 (3)	0.125 (3)	0.0568 (16)	−0.004 (2)	0.0180 (17)	−0.0088 (17)
C35	0.0752 (19)	0.096 (2)	0.125 (3)	0.0177 (17)	0.0214 (19)	0.029 (2)
C36	0.083 (2)	0.082 (2)	0.122 (3)	0.0082 (16)	0.0244 (19)	0.017 (2)
C37	0.0586 (17)	0.094 (2)	0.173 (4)	−0.0089 (16)	−0.001 (2)	0.011 (3)
C2	0.107 (3)	0.090 (2)	0.090 (2)	0.021 (2)	−0.0157 (19)	−0.0022 (18)
C3	0.071 (2)	0.104 (3)	0.144 (4)	−0.0018 (19)	−0.035 (2)	0.010 (3)
C4	0.133 (4)	0.077 (2)	0.181 (5)	0.006 (2)	0.069 (4)	0.020 (3)
C5	0.107 (3)	0.133 (3)	0.085 (2)	0.030 (3)	−0.007 (2)	−0.005 (2)
C6	0.169 (5)	0.0654 (19)	0.118 (3)	−0.001 (2)	−0.002 (3)	−0.0022 (19)
C7	0.080 (2)	0.139 (4)	0.149 (4)	0.009 (2)	0.029 (2)	0.008 (3)
C8	0.091 (3)	0.117 (3)	0.184 (5)	−0.023 (2)	0.050 (3)	0.004 (3)
C9	0.170 (5)	0.110 (3)	0.108 (3)	−0.014 (3)	0.032 (3)	−0.035 (3)
C10	0.118 (3)	0.125 (4)	0.206 (6)	−0.018 (3)	0.067 (4)	0.024 (4)
C11	0.201 (7)	0.228 (8)	0.104 (4)	−0.002 (6)	0.050 (4)	0.012 (4)
C38	0.102 (3)	0.209 (7)	0.226 (7)	−0.006 (4)	0.061 (4)	0.044 (6)

### Tetrabutylammonium chloride–1,1'-(1,2-phenylene)bis(3-m-tolylurea) (1/1) (I) Geometric parameters (Å, º)

**Table:**

O1—C12	1.217 (3)	C8—H8B	0.960 (16)
O2—C13	1.218 (3)	C8—H8C	0.96 (2)
N1—C1	1.391 (3)	C16—H16	0.930 (4)
N1—C13	1.364 (3)	C18—H18	0.93 (4)
N2—C12	1.374 (3)	C19—H19	0.929 (4)
N2—C15	1.422 (3)	C23—H23	0.931 (4)
N3—C12	1.365 (3)	C25—H25	0.930 (6)
N3—C14	1.403 (3)	C27—H27	0.930 (4)
N4—C13	1.359 (3)	C28—H28	0.930 (5)
N4—C17	1.418 (3)	C29—H29	0.929 (4)
N5—C20	1.517 (3)	C30—H30	0.930 (4)
N5—C22	1.522 (4)	C32—H32	0.930 (5)
N5—C24	1.517 (4)	C34—H34A	0.959 (15)
N5—C26	1.514 (3)	C34—H34B	0.96 (2)
C1—C15	1.400 (3)	C34—H34C	0.959 (13)
C1—C18	1.398 (4)	C37—H37	0.930 (6)
C14—C16	1.386 (3)	C2—H2A	0.970 (6)
C14—C19	1.381 (3)	C2—H2B	0.969 (6)
C15—C23	1.381 (4)	C4—H4A	0.971 (6)
C16—C21	1.386 (4)	C4—H4B	0.970 (8)
C17—C25	1.388 (5)	C5—H5A	0.971 (8)
C17—C32	1.381 (4)	C5—H5B	0.969 (7)
C18—C29	1.376 (5)	C6—H6A	0.970 (6)
C19—C28	1.386 (4)	C6—H6B	0.970 (8)
C20—C2	1.520 (5)	C7—H7A	0.971 (8)
C21—C27	1.384 (5)	C7—H7B	0.971 (8)
C21—C34	1.493 (4)	C9—H9A	0.96 (2)
C22—C33	1.500 (4)	C9—H9B	0.959 (11)
C23—C30	1.385 (4)	C9—H9C	0.96 (3)
C24—C36	1.519 (4)	C10—H10A	0.960 (11)
C25—C31	1.385 (4)	C10—H10B	0.96 (3)
C26—C35	1.499 (5)	C10—H10C	0.96 (3)
C27—C28	1.363 (5)	C11—H11A	0.96 (2)
C29—C30	1.370 (5)	C11—H11B	0.96 (3)
C31—C37	1.388 (6)	C11—H11C	0.96 (3)
C31—C8	1.513 (6)	C20—H20A	0.970 (4)
C32—C3	1.408 (5)	C20—H20B	0.970 (4)
C33—C6	1.489 (5)	C22—H22A	0.970 (4)
C35—C7	1.467 (5)	C22—H22B	0.970 (4)
C36—C4	1.482 (5)	C24—H24A	0.970 (4)
C37—C3	1.360 (7)	C24—H24B	0.971 (4)
C2—C5	1.515 (5)	C26—H26A	0.971 (4)
C4—C10	1.514 (6)	C26—H26B	0.970 (4)
C5—C11	1.445 (8)	C33—H33A	0.970 (5)
C6—C9	1.433 (6)	C33—H33B	0.970 (6)
C7—C38	1.465 (7)	C35—H35A	0.969 (6)
N1—H1	0.860 (3)	C35—H35B	0.969 (6)
N2—H2	0.860 (3)	C36—H36A	0.970 (6)
N3—H3	0.860 (3)	C36—H36B	0.971 (6)
N4—H4	0.860 (3)	C38—H38A	0.96 (4)
C3—H3A	0.931 (8)	C38—H38B	0.96 (3)
C8—H8A	0.96 (3)	C38—H38C	0.96 (4)
			
C13—N1—C1	127.1 (2)	C29—C30—H30	120.3 (3)
C15—N2—C12	122.13 (18)	C3—C32—H32	121.1 (5)
C14—N3—C12	128.19 (19)	C17—C32—H32	121.3 (4)
C17—N4—C13	128.1 (2)	C21—C34—H34A	109.4 (3)
C22—N5—C20	105.98 (19)	C21—C34—H34B	109.4 (9)
C24—N5—C20	111.0 (2)	C21—C34—H34C	109.5 (9)
C24—N5—C22	110.9 (2)	H34A—C34—H34B	110 (3)
C26—N5—C20	110.9 (2)	H34A—C34—H34C	109 (3)
C26—N5—C22	111.2 (2)	H34B—C34—H34C	109.5 (17)
C26—N5—C24	106.91 (19)	C5—C2—H2A	109.7 (5)
C15—C1—N1	118.8 (2)	C5—C2—H2B	109.8 (5)
C18—C1—N1	122.7 (2)	C20—C2—H2A	109.7 (4)
C18—C1—C15	118.3 (2)	C20—C2—H2B	109.8 (4)
N2—C12—O1	123.7 (2)	H2A—C2—H2B	108.2 (5)
N3—C12—O1	124.7 (2)	C10—C4—H4A	108.8 (6)
N3—C12—N2	111.66 (18)	C10—C4—H4B	108.8 (6)
N1—C13—O2	124.0 (2)	C36—C4—H4A	108.8 (6)
N4—C13—O2	124.6 (2)	C36—C4—H4B	108.8 (5)
N4—C13—N1	111.4 (2)	H4A—C4—H4B	107.6 (6)
C16—C14—N3	116.9 (2)	C2—C5—H5A	108.6 (6)
C19—C14—N3	123.8 (2)	C2—C5—H5B	108.6 (5)
C19—C14—C16	119.3 (2)	C11—C5—H5A	108.7 (5)
C1—C15—N2	121.1 (2)	C11—C5—H5B	108.7 (6)
C23—C15—N2	118.4 (2)	H5A—C5—H5B	107.5 (6)
C23—C15—C1	120.3 (2)	C9—C6—H6A	107.5 (5)
H16—C16—C14	119.18 (15)	C9—C6—H6B	107.5 (5)
C21—C16—C14	121.6 (3)	C33—C6—H6A	107.5 (4)
C25—C17—N4	115.9 (3)	C33—C6—H6B	107.6 (5)
C32—C17—N4	123.9 (3)	H6A—C6—H6B	107.0 (6)
C32—C17—C25	120.3 (3)	C35—C7—H7A	108.5 (5)
C29—C18—C1	120.2 (3)	C35—C7—H7B	108.4 (5)
C28—C19—C14	118.9 (3)	C38—C7—H7A	108.4 (5)
C2—C20—N5	115.8 (2)	C38—C7—H7B	108.3 (6)
C27—C21—C16	118.2 (3)	H7A—C7—H7B	107.3 (7)
C34—C21—C16	120.6 (3)	C6—C9—H9A	109.4 (9)
C34—C21—C27	121.1 (3)	C6—C9—H9B	109.7 (14)
C33—C22—N5	115.8 (2)	C6—C9—H9C	109.6 (13)
H23—C23—C15	119.77 (16)	H9A—C9—H9B	109 (3)
C30—C23—C15	120.5 (3)	H9A—C9—H9C	109 (3)
C36—C24—N5	115.5 (2)	H9B—C9—H9C	110 (3)
C31—C25—C17	121.7 (3)	C4—C10—H10A	109.5 (8)
C35—C26—N5	115.7 (2)	C4—C10—H10B	109.5 (14)
H27—C27—C21	119.82 (17)	C4—C10—H10C	109.5 (10)
C28—C27—C21	120.4 (3)	H10A—C10—H10B	109 (4)
C27—C28—C19	121.6 (3)	H10A—C10—H10C	110 (4)
C30—C29—C18	121.3 (3)	H10B—C10—H10C	109 (3)
C29—C30—C23	119.3 (3)	C5—C11—H11A	109 (2)
C37—C31—C25	117.7 (4)	C5—C11—H11B	110 (3)
C8—C31—C25	120.4 (4)	C5—C11—H11C	110 (2)
C8—C31—C37	121.9 (4)	H11A—C11—H11B	110 (4)
C3—C32—C17	117.6 (4)	H11A—C11—H11C	109 (4)
C6—C33—C22	112.1 (3)	H11B—C11—H11C	110 (4)
C7—C35—C26	112.1 (3)	N5—C20—H20A	108.3 (3)
C4—C36—C24	111.8 (3)	N5—C20—H20B	108.3 (3)
C3—C37—C31	121.1 (3)	C2—C20—H20A	108.3 (3)
C5—C2—C20	109.7 (3)	C2—C20—H20B	108.4 (3)
C37—C3—C32	121.5 (4)	H20A—C20—H20B	107.4 (4)
C10—C4—C36	113.7 (4)	N5—C22—H22A	108.3 (3)
C11—C5—C2	114.5 (5)	N5—C22—H22B	108.3 (3)
C9—C6—C33	119.0 (4)	C33—C22—H22A	108.4 (3)
C38—C7—C35	115.6 (5)	C33—C22—H22B	108.3 (3)
C1—N1—H1	116.5 (3)	H22A—C22—H22B	107.4 (3)
C13—N1—H1	116.5 (3)	N5—C24—H24A	108.4 (3)
C12—N2—H2	118.9 (2)	N5—C24—H24B	108.4 (3)
C15—N2—H2	118.9 (2)	C36—C24—H24A	108.5 (3)
C12—N3—H3	115.9 (2)	C36—C24—H24B	108.4 (3)
C14—N3—H3	115.9 (2)	H24A—C24—H24B	107.5 (4)
C13—N4—H4	115.9 (3)	N5—C26—H26A	108.4 (3)
C17—N4—H4	116.0 (3)	N5—C26—H26B	108.3 (3)
C32—C3—H3A	119.2 (5)	C35—C26—H26A	108.4 (3)
C37—C3—H3A	119.2 (5)	C35—C26—H26B	108.4 (3)
C31—C8—H8A	109.5 (14)	H26A—C26—H26B	107.4 (4)
C31—C8—H8B	109.7 (18)	C6—C33—H33A	109.2 (4)
C31—C8—H8C	109.5 (7)	C6—C33—H33B	109.2 (4)
H8A—C8—H8B	110 (2)	C22—C33—H33A	109.3 (4)
H8A—C8—H8C	109 (3)	C22—C33—H33B	109.2 (3)
H8B—C8—H8C	110 (3)	H33A—C33—H33B	107.9 (4)
C14—C16—H16	119.2 (3)	C7—C35—H35A	109.2 (5)
C21—C16—H16	119.2 (3)	C7—C35—H35B	109.2 (5)
C1—C18—H18	120 (4)	C26—C35—H35A	109.2 (4)
C29—C18—H18	119.8 (17)	C26—C35—H35B	109.1 (4)
C14—C19—H19	120.6 (3)	H35A—C35—H35B	108.0 (5)
C28—C19—H19	120.5 (3)	C4—C36—H36A	109.2 (5)
C15—C23—H23	119.7 (3)	C4—C36—H36B	109.2 (5)
C30—C23—H23	119.8 (3)	C24—C36—H36A	109.3 (4)
C17—C25—H25	119.1 (3)	C24—C36—H36B	109.3 (4)
C31—C25—H25	119.2 (4)	H36A—C36—H36B	107.9 (5)
C21—C27—H27	119.9 (3)	C7—C38—H38A	109.7 (15)
C28—C27—H27	119.8 (4)	C7—C38—H38B	109 (2)
C19—C28—H28	119.2 (5)	C7—C38—H38C	109.5 (16)
C27—C28—H28	119.2 (4)	H38A—C38—H38B	109 (3)
C18—C29—H29	119.3 (7)	H38A—C38—H38C	110 (4)
C30—C29—H29	119.4 (5)	H38B—C38—H38C	109 (4)
C23—C30—H30	120.4 (3)		

### Tetrabutylammonium chloride–1,1'-(1,2-phenylene)bis(3-m-tolylurea) (1/1) (I) Hydrogen-bond geometry (Å, º)

**Table:**

*D*—H···*A*	*D*—H	H···*A*	*D*···*A*	*D*—H···*A*
N1—H1···Cl1	0.86 (1)	2.53 (1)	3.348 (2)	159 (1)
N2^i^—H2^i^···Cl1	0.86 (1)	2.62 (1)	3.231 (2)	129 (1)
N3^i^—H3^i^···Cl1	0.86 (1)	2.55 (1)	3.285 (2)	144 (1)
N4—H4···Cl1	0.86 (1)	2.34 (1)	3.191 (2)	169 (1)
C26—H26*a*···O1	0.97 (1)	2.38 (1)	3.307 (3)	159 (1)
C22^ii^—H22*a*^ii^···Cl1	0.97 (1)	3.05 (1)	3.938 (3)	152 (1)
C20^ii^—H20*b*^ii^···Cl1	0.97 (1)	3.11 (1)	3.984 (3)	150 (1)

Symmetry codes: (i) −*x*+2, −*y*+1, −*z*+1; (ii) −*x*+3/2, *y*−1/2, −*z*+1/2.

### Tetrabutylammonium bromide–1,1'-(1,2-phenylene)bis(3-m-tolylurea) (1/1) (II) Crystal data

**Table:**

C_16_H_36_N^+^·Br^−^·C_22_H_22_N_4_O_2_	*F*(000) = 1489.0393
*M**_r_* = 696.80	*D*_x_ = 1.190 Mg m^−^^3^
Monoclinic, *P*2_1_/*c*	Cu *K*α radiation, λ = 1.54184 Å
*a* = 10.5879 (2) Å	Cell parameters from 7946 reflections
*b* = 20.3165 (5) Å	θ = 4.7–72.6°
*c* = 18.0828 (3) Å	µ = 1.72 mm^−^^1^
β = 91.0672 (17)°	*T* = 294 K
*V* = 3889.11 (14) Å^3^	Plate, clear light colourless
*Z* = 4	0.5 × 0.3 × 0.2 mm

### Tetrabutylammonium bromide–1,1'-(1,2-phenylene)bis(3-m-tolylurea) (1/1) (II) Data collection

**Table:**

Agilent New Gemini, Dual, Cu at zero, EosS2 diffractometer	6081 reflections with *I*≥ 2u(*I*)
Detector resolution: 15.9595 pixels mm^-1^	*R*_int_ = 0.039
ω scans	θ_max_ = 72.5°, θ_min_ = 4.4°
Absorption correction: multi-scan (CrysAlis PRO; Agilent, 2014)	*h* = −8→13
*T*_min_ = 0.444, *T*_max_ = 1.000	*k* = −25→22
21853 measured reflections	*l* = −22→19
7582 independent reflections	

### Tetrabutylammonium bromide–1,1'-(1,2-phenylene)bis(3-m-tolylurea) (1/1) (II) Refinement

**Table:**

Refinement on *F*^2^	0 restraints
Least-squares matrix: full	92 constraints
*R*[*F*^2^ > 2σ(*F*^2^)] = 0.063	H-atom parameters constrained
*wR*(*F*^2^) = 0.185	*w* = 1/[σ^2^(*F*_o_^2^) + (0.1213*P*)^2^ + 0.5505*P*] where *P* = (*F*_o_^2^ + 2*F*_c_^2^)/3
*S* = 1.03	(Δ/σ)_max_ = 0.003
7582 reflections	Δρ_max_ = 1.02 e Å^−^^3^
421 parameters	Δρ_min_ = −0.54 e Å^−^^3^

### Tetrabutylammonium bromide–1,1'-(1,2-phenylene)bis(3-m-tolylurea) (1/1) (II) Fractional atomic coordinates and isotropic or equivalent isotropic displacement parameters (Å^2^)

**Table:**

	*x*	*y*	*z*	*U*_iso_*/*U*_eq_	
Br1	0.34211 (3)	0.436876 (19)	0.380770 (15)	0.06780 (16)	
O1	0.5943 (2)	0.53896 (12)	0.16204 (11)	0.0652 (5)	
O2	0.5000 (2)	0.63426 (12)	0.36423 (12)	0.0708 (6)	
N1	0.5894 (2)	0.51747 (12)	0.28578 (12)	0.0553 (5)	
H1	0.5429 (2)	0.50181 (12)	0.32004 (12)	0.0664 (6)*	
N2	0.4204 (2)	0.49196 (13)	0.21256 (12)	0.0594 (6)	
H2	0.3891 (2)	0.48089 (13)	0.25431 (12)	0.0713 (7)*	
N3	0.6278 (2)	0.56443 (11)	0.43059 (12)	0.0551 (5)	
H3	0.6325 (2)	0.53992 (11)	0.46931 (12)	0.0662 (6)*	
N4	0.4466 (2)	0.60268 (12)	0.48120 (12)	0.0580 (5)	
H4	0.4770 (2)	0.58314 (12)	0.51979 (12)	0.0696 (6)*	
N5	0.4877 (2)	0.73056 (13)	0.16681 (14)	0.0641 (6)	
C1	0.5396 (3)	0.51776 (13)	0.21594 (14)	0.0509 (5)	
C12	0.7096 (3)	0.54029 (14)	0.30718 (14)	0.0534 (6)	
C13	0.5225 (3)	0.60358 (13)	0.42087 (14)	0.0531 (6)	
C14	0.7283 (3)	0.56240 (13)	0.38032 (16)	0.0550 (6)	
C15	0.3253 (3)	0.62969 (13)	0.48807 (15)	0.0556 (6)	
C16	0.3425 (3)	0.48110 (14)	0.15060 (14)	0.0530 (6)	
C17	0.2207 (3)	0.45888 (15)	0.16407 (16)	0.0578 (6)	
H17	0.1967 (3)	0.45104 (15)	0.21250 (16)	0.0693 (7)*	
C18	0.8125 (3)	0.53723 (17)	0.26118 (17)	0.0656 (7)	
H18	0.8009 (3)	0.52385 (17)	0.21234 (17)	0.0787 (9)*	
C19	0.2560 (3)	0.60972 (15)	0.54806 (18)	0.0654 (7)	
H19	0.2911 (3)	0.57941 (15)	0.58105 (18)	0.0785 (9)*	
C20	0.3884 (3)	0.67697 (15)	0.16932 (18)	0.0665 (7)	
H20a	0.4245 (3)	0.63682 (15)	0.14980 (18)	0.0798 (9)*	
H20b	0.3684 (3)	0.66885 (15)	0.22065 (18)	0.0798 (9)*	
C21	0.8496 (3)	0.57762 (17)	0.40527 (19)	0.0686 (8)	
H21	0.8624 (3)	0.59108 (17)	0.45399 (19)	0.0824 (9)*	
C22	0.1347 (3)	0.44823 (17)	0.10692 (19)	0.0664 (7)	
C23	0.5244 (3)	0.74585 (17)	0.08761 (18)	0.0704 (8)	
H23a	0.4507 (3)	0.76289 (17)	0.06133 (18)	0.0845 (10)*	
H23b	0.5877 (3)	0.78041 (17)	0.08871 (18)	0.0845 (10)*	
C24	0.6001 (3)	0.70568 (16)	0.21146 (18)	0.0661 (7)	
H24a	0.5718 (3)	0.69477 (16)	0.26068 (18)	0.0793 (9)*	
H24b	0.6294 (3)	0.66527 (16)	0.18893 (18)	0.0793 (9)*	
C25	0.3791 (3)	0.49119 (18)	0.07795 (16)	0.0670 (8)	
H25	0.4603 (3)	0.50570 (18)	0.06778 (16)	0.0804 (9)*	
C26	0.1355 (3)	0.63368 (17)	0.5604 (2)	0.0768 (9)	
C27	0.4370 (3)	0.79435 (16)	0.1983 (2)	0.0734 (8)	
H27a	0.5024 (3)	0.82761 (16)	0.1951 (2)	0.0881 (10)*	
H27b	0.3664 (3)	0.80872 (16)	0.1673 (2)	0.0881 (10)*	
C28	0.1728 (3)	0.4587 (2)	0.03532 (19)	0.0754 (9)	
H28	0.1160 (3)	0.4518 (2)	−0.00377 (19)	0.0905 (10)*	
C29	0.9512 (3)	0.57316 (19)	0.3592 (2)	0.0786 (10)	
H29	1.0321 (3)	0.58316 (19)	0.3767 (2)	0.0943 (11)*	
C30	0.9318 (3)	0.5536 (2)	0.2865 (2)	0.0777 (9)	
H30	0.9997 (3)	0.5516 (2)	0.2546 (2)	0.0932 (11)*	
C31	0.2928 (3)	0.4793 (2)	0.02111 (17)	0.0781 (9)	
H31	0.3170 (3)	0.4853 (2)	−0.02761 (17)	0.0937 (11)*	
C32	0.2736 (3)	0.67575 (19)	0.4405 (2)	0.0770 (9)	
H32	0.3188 (3)	0.69045 (19)	0.4000 (2)	0.0924 (11)*	
C33	0.2667 (3)	0.69113 (19)	0.1273 (2)	0.0822 (10)	
H33a	0.2353 (3)	0.73411 (19)	0.1413 (2)	0.0987 (12)*	
H33b	0.2828 (3)	0.69188 (19)	0.0746 (2)	0.0987 (12)*	
C34	0.5761 (4)	0.6878 (2)	0.0441 (2)	0.0837 (10)	
H34a	0.6413 (4)	0.6658 (2)	0.0732 (2)	0.1004 (12)*	
H34b	0.5089 (4)	0.6565 (2)	0.0340 (2)	0.1004 (12)*	
C35	0.0860 (3)	0.6795 (2)	0.5119 (2)	0.0832 (10)	
H35	0.0057 (3)	0.6966 (2)	0.5193 (2)	0.0999 (12)*	
C36	0.3937 (4)	0.79121 (19)	0.2773 (2)	0.0885 (11)	
H36a	0.3246 (4)	0.76006 (19)	0.2804 (2)	0.1062 (13)*	
H36b	0.4625 (4)	0.77515 (19)	0.3084 (2)	0.1062 (13)*	
C37	0.7107 (4)	0.7520 (2)	0.2190 (3)	0.0901 (12)	
H37a	0.7391 (4)	0.7644 (2)	0.1702 (3)	0.1081 (14)*	
H37b	0.6844 (4)	0.7917 (2)	0.2442 (3)	0.1081 (14)*	
C2	0.6311 (4)	0.7119 (3)	−0.0290 (2)	0.0922 (12)	
H2a	0.6438 (4)	0.6744 (3)	−0.0611 (2)	0.1106 (14)*	
H2b	0.5706 (4)	0.7410 (3)	−0.0532 (2)	0.1106 (14)*	
C3	0.0019 (4)	0.4253 (2)	0.1226 (3)	0.0939 (13)	
H3a	−0.0115 (13)	0.3826 (9)	0.1012 (19)	0.1409 (19)*	
H3b	−0.0092 (12)	0.4229 (19)	0.1751 (3)	0.1409 (19)*	
H3c	−0.0577 (4)	0.4559 (10)	0.1015 (19)	0.1409 (19)*	
C4	0.1542 (4)	0.6998 (2)	0.4534 (3)	0.0915 (11)	
H4a	0.1195 (4)	0.7308 (2)	0.4212 (3)	0.1099 (14)*	
C5	0.8161 (4)	0.7213 (3)	0.2608 (3)	0.1035 (14)	
H5a	0.7852 (4)	0.7068 (3)	0.3083 (3)	0.1241 (17)*	
H5b	0.8433 (4)	0.6825 (3)	0.2342 (3)	0.1241 (17)*	
C6	0.1691 (4)	0.6399 (2)	0.1432 (3)	0.0967 (12)	
H6a	0.2039 (4)	0.5969 (2)	0.1322 (3)	0.1161 (15)*	
H6b	0.1511 (4)	0.6410 (2)	0.1956 (3)	0.1161 (15)*	
C7	0.3513 (6)	0.8563 (2)	0.3056 (3)	0.1212 (19)	
H7a	0.4176 (6)	0.8880 (2)	0.2963 (3)	0.145 (2)*	
H7b	0.2779 (6)	0.8698 (2)	0.2764 (3)	0.145 (2)*	
C8	0.0478 (5)	0.6480 (3)	0.1005 (3)	0.1172 (17)	
H8a	0.0640 (8)	0.646 (2)	0.0485 (3)	0.176 (3)*	
H8b	−0.0095 (17)	0.6135 (14)	0.114 (2)	0.176 (3)*	
H8c	0.011 (2)	0.6899 (10)	0.112 (2)	0.176 (3)*	
C9	0.3190 (5)	0.8613 (3)	0.3830 (3)	0.1225 (19)	
H9a	0.243 (3)	0.8368 (19)	0.3916 (6)	0.184 (3)*	
H9b	0.387 (2)	0.844 (2)	0.4130 (3)	0.184 (3)*	
H9c	0.306 (4)	0.9067 (4)	0.3955 (7)	0.184 (3)*	
C10	0.0624 (5)	0.6099 (3)	0.6255 (4)	0.127 (2)	
H10a	0.057 (4)	0.5628 (4)	0.6241 (16)	0.191 (3)*	
H10b	0.105 (3)	0.623 (2)	0.6704 (4)	0.191 (3)*	
H10c	−0.0211 (17)	0.628 (2)	0.6235 (16)	0.191 (3)*	
C11	0.9261 (6)	0.7642 (4)	0.2737 (4)	0.145 (3)	
H11a	0.961 (3)	0.777 (2)	0.2271 (4)	0.217 (4)*	
H11b	0.9004 (12)	0.8030 (14)	0.300 (3)	0.217 (4)*	
H11c	0.989 (2)	0.7411 (11)	0.303 (3)	0.217 (4)*	
C38	0.7528 (5)	0.7474 (3)	−0.0189 (3)	0.133 (2)	
H38a	0.775 (3)	0.768 (2)	−0.0646 (10)	0.199 (3)*	
H38b	0.7445 (17)	0.780 (2)	0.019 (2)	0.199 (3)*	
H38c	0.8177 (13)	0.7167 (6)	−0.004 (3)	0.199 (3)*	

### Tetrabutylammonium bromide–1,1'-(1,2-phenylene)bis(3-m-tolylurea) (1/1) (II) Atomic displacement parameters (Å^2^)

**Table:**

	*U*^11^	*U*^22^	*U*^33^	*U*^12^	*U*^13^	*U*^23^
Br1	0.0685 (2)	0.0906 (3)	0.0443 (2)	−0.00528 (15)	0.00192 (13)	0.00556 (13)
O1	0.0731 (12)	0.0763 (13)	0.0464 (10)	−0.0165 (10)	0.0042 (9)	0.0070 (9)
O2	0.0846 (14)	0.0732 (13)	0.0547 (11)	0.0110 (11)	0.0046 (10)	0.0153 (10)
N1	0.0561 (12)	0.0684 (14)	0.0417 (10)	−0.0119 (10)	0.0055 (9)	−0.0039 (9)
N2	0.0575 (12)	0.0815 (16)	0.0392 (11)	−0.0067 (11)	−0.0001 (9)	0.0050 (10)
N3	0.0647 (13)	0.0601 (13)	0.0406 (11)	0.0002 (10)	−0.0003 (9)	0.0002 (9)
N4	0.0658 (13)	0.0605 (13)	0.0476 (11)	0.0095 (10)	0.0011 (9)	0.0053 (9)
N5	0.0708 (14)	0.0549 (13)	0.0666 (14)	0.0003 (11)	−0.0030 (11)	0.0210 (11)
C1	0.0607 (14)	0.0500 (13)	0.0422 (12)	−0.0001 (11)	0.0041 (10)	−0.0009 (10)
C12	0.0585 (14)	0.0542 (14)	0.0475 (13)	−0.0084 (11)	0.0040 (11)	−0.0006 (10)
C13	0.0642 (14)	0.0502 (13)	0.0448 (12)	−0.0027 (11)	−0.0005 (10)	−0.0006 (10)
C14	0.0609 (15)	0.0538 (14)	0.0501 (14)	−0.0057 (11)	−0.0006 (11)	−0.0023 (10)
C15	0.0596 (14)	0.0497 (13)	0.0572 (14)	−0.0014 (11)	−0.0061 (11)	−0.0071 (11)
C16	0.0568 (13)	0.0568 (14)	0.0452 (12)	0.0038 (11)	−0.0006 (10)	0.0030 (10)
C17	0.0600 (15)	0.0628 (16)	0.0505 (14)	−0.0009 (12)	−0.0021 (11)	0.0068 (12)
C18	0.0637 (16)	0.0745 (19)	0.0588 (16)	−0.0063 (14)	0.0106 (13)	−0.0065 (14)
C19	0.0755 (18)	0.0513 (15)	0.0697 (18)	0.0041 (13)	0.0088 (14)	−0.0042 (13)
C20	0.0740 (18)	0.0558 (16)	0.0699 (18)	−0.0020 (13)	0.0038 (14)	0.0163 (13)
C21	0.0664 (18)	0.0737 (19)	0.0655 (17)	−0.0101 (14)	−0.0082 (14)	−0.0109 (15)
C22	0.0630 (16)	0.0685 (18)	0.0675 (18)	−0.0026 (13)	−0.0073 (14)	0.0038 (14)
C23	0.0761 (19)	0.0717 (19)	0.0633 (17)	−0.0025 (15)	−0.0038 (14)	0.0268 (14)
C24	0.0748 (17)	0.0603 (16)	0.0630 (16)	0.0036 (14)	−0.0033 (14)	0.0187 (13)
C25	0.0639 (16)	0.089 (2)	0.0482 (14)	−0.0047 (15)	−0.0003 (12)	0.0054 (14)
C26	0.0728 (19)	0.0630 (18)	0.095 (2)	−0.0017 (15)	0.0122 (17)	−0.0199 (17)
C27	0.082 (2)	0.0520 (16)	0.086 (2)	−0.0013 (14)	−0.0037 (16)	0.0170 (15)
C28	0.0743 (19)	0.092 (2)	0.0589 (17)	−0.0072 (17)	−0.0177 (15)	0.0042 (16)
C29	0.0558 (17)	0.087 (2)	0.093 (2)	−0.0149 (15)	−0.0044 (16)	−0.0094 (18)
C30	0.0611 (18)	0.090 (2)	0.083 (2)	−0.0126 (16)	0.0162 (16)	−0.0030 (18)
C31	0.083 (2)	0.108 (3)	0.0429 (14)	−0.0101 (19)	−0.0024 (13)	0.0070 (15)
C32	0.0753 (19)	0.080 (2)	0.075 (2)	0.0067 (16)	−0.0106 (16)	0.0119 (17)
C33	0.077 (2)	0.074 (2)	0.095 (3)	−0.0052 (17)	−0.0055 (18)	0.0197 (19)
C34	0.101 (3)	0.085 (2)	0.0650 (19)	−0.007 (2)	−0.0007 (17)	0.0179 (17)
C35	0.0585 (17)	0.081 (2)	0.110 (3)	0.0073 (16)	−0.0114 (18)	−0.022 (2)
C36	0.115 (3)	0.0616 (19)	0.089 (2)	0.0140 (19)	0.008 (2)	0.0103 (17)
C37	0.095 (3)	0.074 (2)	0.100 (3)	−0.0102 (19)	−0.025 (2)	0.022 (2)
C2	0.091 (2)	0.118 (3)	0.067 (2)	−0.005 (2)	0.0016 (18)	0.019 (2)
C3	0.066 (2)	0.125 (4)	0.090 (3)	−0.017 (2)	−0.0103 (18)	0.008 (2)
C4	0.079 (2)	0.090 (3)	0.104 (3)	0.017 (2)	−0.030 (2)	0.003 (2)
C5	0.101 (3)	0.108 (3)	0.101 (3)	−0.011 (3)	−0.030 (2)	0.023 (3)
C6	0.085 (2)	0.076 (2)	0.130 (4)	−0.0086 (19)	−0.002 (2)	0.016 (2)
C7	0.160 (5)	0.062 (2)	0.143 (5)	0.013 (3)	0.027 (4)	−0.000 (3)
C8	0.091 (3)	0.135 (4)	0.125 (4)	−0.032 (3)	−0.013 (3)	0.000 (3)
C9	0.114 (4)	0.109 (4)	0.144 (5)	0.030 (3)	−0.002 (3)	−0.045 (3)
C10	0.114 (4)	0.121 (4)	0.149 (5)	0.022 (3)	0.062 (4)	0.003 (4)
C11	0.120 (4)	0.163 (6)	0.149 (5)	−0.044 (4)	−0.054 (4)	0.030 (5)
C38	0.108 (4)	0.179 (7)	0.112 (4)	−0.040 (4)	0.013 (3)	0.019 (4)

### Tetrabutylammonium bromide–1,1'-(1,2-phenylene)bis(3-m-tolylurea) (1/1) (II) Geometric parameters (Å, º)

**Table:**

O1—C1	1.222 (3)	C3—H3B	0.960 (8)
O2—C13	1.219 (3)	C3—H3C	0.960 (19)
N1—C1	1.360 (3)	C4—H4A	0.929 (7)
N1—C12	1.402 (3)	C10—H10A	0.959 (10)
N2—C1	1.367 (3)	C10—H10B	0.96 (2)
N2—C16	1.397 (3)	C10—H10C	0.96 (2)
N3—C13	1.378 (4)	C17—H17	0.930 (4)
N3—C14	1.413 (4)	C18—H18	0.930 (4)
N4—C13	1.367 (4)	C19—H19	0.930 (4)
N4—C15	1.405 (4)	C21—H21	0.930 (5)
N5—C20	1.515 (4)	C25—H25	0.931 (5)
N5—C23	1.523 (4)	C28—H28	0.930 (5)
N5—C24	1.513 (4)	C29—H29	0.930 (5)
N5—C27	1.518 (4)	C30—H30	0.931 (5)
C12—C14	1.408 (4)	C31—H31	0.930 (4)
C12—C18	1.385 (4)	C2—H2A	0.969 (7)
C14—C21	1.387 (4)	C2—H2B	0.970 (7)
C15—C19	1.382 (4)	C5—H5A	0.971 (8)
C15—C32	1.378 (4)	C5—H5B	0.970 (8)
C16—C17	1.392 (4)	C6—H6A	0.970 (6)
C16—C25	1.392 (4)	C6—H6B	0.970 (8)
C17—C22	1.382 (4)	C7—H7A	0.970 (8)
C18—C30	1.376 (5)	C7—H7B	0.971 (8)
C19—C26	1.387 (5)	C8—H8A	0.960 (8)
C20—C33	1.511 (5)	C8—H8B	0.96 (3)
C21—C29	1.376 (5)	C8—H8C	0.96 (2)
C22—C28	1.380 (5)	C9—H9A	0.96 (3)
C22—C3	1.512 (5)	C9—H9B	0.96 (2)
C23—C34	1.524 (5)	C9—H9C	0.960 (12)
C24—C37	1.507 (5)	C11—H11A	0.96 (2)
C25—C31	1.384 (4)	C11—H11B	0.96 (4)
C26—C35	1.376 (6)	C11—H11C	0.97 (4)
C26—C10	1.500 (6)	C20—H20A	0.970 (4)
C27—C36	1.509 (5)	C20—H20B	0.970 (5)
C28—C31	1.367 (5)	C23—H23A	0.970 (5)
C29—C30	1.385 (6)	C23—H23B	0.971 (5)
C32—C4	1.380 (5)	C24—H24A	0.970 (5)
C33—C6	1.498 (5)	C24—H24B	0.970 (5)
C34—C2	1.534 (5)	C27—H27A	0.970 (5)
C35—C4	1.356 (6)	C27—H27B	0.971 (5)
C36—C7	1.491 (6)	C33—H33A	0.970 (5)
C37—C5	1.474 (5)	C33—H33B	0.971 (5)
C2—C38	1.485 (6)	C34—H34A	0.969 (6)
C5—C11	1.470 (7)	C34—H34B	0.969 (6)
C6—C8	1.495 (6)	C36—H36A	0.970 (6)
C7—C9	1.450 (7)	C36—H36B	0.969 (6)
N1—H1	0.860 (3)	C37—H37A	0.971 (7)
N2—H2	0.860 (3)	C37—H37B	0.970 (6)
N3—H3	0.860 (3)	C38—H38A	0.96 (3)
N4—H4	0.860 (3)	C38—H38B	0.96 (4)
C3—H3A	0.96 (2)	C38—H38C	0.96 (2)
			
C12—N1—C1	126.1 (2)	C30—C29—H29	120.3 (4)
C16—N2—C1	128.9 (2)	C18—C30—H30	119.9 (4)
C14—N3—C13	123.5 (2)	C29—C30—H30	119.9 (4)
C15—N4—C13	128.1 (2)	C25—C31—H31	119.4 (4)
C23—N5—C20	111.4 (3)	C28—C31—H31	119.5 (4)
C24—N5—C20	106.5 (2)	C4—C32—H32	120.4 (4)
C24—N5—C23	111.0 (2)	C15—C32—H32	120.3 (4)
C27—N5—C20	110.6 (2)	C4—C35—H35	120.0 (5)
C27—N5—C23	106.1 (2)	C26—C35—H35	120.0 (4)
C27—N5—C24	111.4 (3)	C34—C2—H2A	108.9 (6)
N1—C1—O1	124.1 (2)	C34—C2—H2B	109.0 (4)
N2—C1—O1	123.4 (2)	C38—C2—H2A	109.0 (5)
N2—C1—N1	112.5 (2)	C38—C2—H2B	108.9 (6)
C14—C12—N1	118.5 (2)	H2A—C2—H2B	107.9 (5)
C18—C12—N1	122.7 (3)	C11—C5—H5A	108.6 (6)
C18—C12—C14	118.7 (3)	C11—C5—H5B	108.6 (5)
N3—C13—O2	123.2 (3)	C37—C5—H5A	108.5 (5)
N4—C13—O2	124.7 (3)	C37—C5—H5B	108.6 (5)
N4—C13—N3	112.1 (2)	H5A—C5—H5B	107.5 (7)
C12—C14—N3	121.2 (3)	C8—C6—H6A	108.6 (5)
C21—C14—N3	119.2 (3)	C8—C6—H6B	108.6 (5)
C21—C14—C12	119.4 (3)	C33—C6—H6A	108.7 (5)
C19—C15—N4	117.0 (3)	C33—C6—H6B	108.7 (5)
C32—C15—N4	124.3 (3)	H6A—C6—H6B	107.7 (6)
C32—C15—C19	118.7 (3)	C9—C7—H7A	107.8 (6)
C17—C16—N2	116.5 (2)	C9—C7—H7B	107.7 (6)
C25—C16—N2	124.3 (3)	C36—C7—H7A	107.8 (6)
C25—C16—C17	119.2 (3)	C36—C7—H7B	107.9 (5)
C22—C17—C16	121.3 (3)	H7A—C7—H7B	107.0 (6)
C30—C18—C12	121.2 (3)	C6—C8—H8A	109.5 (7)
C26—C19—C15	121.8 (3)	C6—C8—H8B	109.2 (17)
C33—C20—N5	115.7 (3)	C6—C8—H8C	109.5 (17)
C29—C21—C14	121.2 (3)	H8A—C8—H8B	110 (3)
C28—C22—C17	118.5 (3)	H8A—C8—H8C	109 (3)
C3—C22—C17	120.6 (3)	H8B—C8—H8C	109 (2)
C3—C22—C28	120.8 (3)	C7—C9—H9A	109.5 (10)
C34—C23—N5	115.3 (3)	C7—C9—H9B	109.4 (10)
C37—C24—N5	116.2 (3)	C7—C9—H9C	109.4 (10)
C31—C25—C16	118.9 (3)	H9A—C9—H9B	110 (3)
C35—C26—C19	118.4 (4)	H9A—C9—H9C	110 (3)
C10—C26—C19	120.2 (4)	H9B—C9—H9C	109 (3)
C10—C26—C35	121.4 (4)	C5—C11—H11A	109.7 (19)
C36—C27—N5	115.8 (3)	C5—C11—H11B	109.5 (11)
C31—C28—C22	120.8 (3)	C5—C11—H11C	109.6 (15)
C30—C29—C21	119.3 (3)	H11A—C11—H11B	109 (4)
C29—C30—C18	120.2 (3)	H11A—C11—H11C	110 (3)
C28—C31—C25	121.1 (3)	H11B—C11—H11C	109 (4)
C4—C32—C15	119.2 (4)	N5—C20—H20A	108.4 (3)
C6—C33—C20	110.9 (3)	N5—C20—H20B	108.4 (3)
C2—C34—C23	110.1 (3)	C33—C20—H20A	108.4 (3)
C4—C35—C26	120.0 (3)	C33—C20—H20B	108.3 (3)
C7—C36—C27	112.8 (4)	H20A—C20—H20B	107.4 (4)
C5—C37—C24	111.1 (3)	N5—C23—H23A	108.5 (3)
C38—C2—C34	113.1 (4)	N5—C23—H23B	108.5 (3)
C35—C4—C32	121.9 (4)	C34—C23—H23A	108.5 (4)
C11—C5—C37	114.8 (5)	C34—C23—H23B	108.4 (4)
C8—C6—C33	114.5 (4)	H23A—C23—H23B	107.5 (4)
C9—C7—C36	118.2 (4)	N5—C24—H24A	108.2 (3)
C1—N1—H1	117.0 (3)	N5—C24—H24B	108.3 (3)
C12—N1—H1	116.9 (3)	C37—C24—H24A	108.2 (4)
C1—N2—H2	115.6 (3)	C37—C24—H24B	108.2 (4)
C16—N2—H2	115.6 (3)	H24A—C24—H24B	107.4 (4)
C13—N3—H3	118.2 (3)	N5—C27—H27A	108.3 (3)
C14—N3—H3	118.3 (3)	N5—C27—H27B	108.3 (4)
C13—N4—H4	115.9 (3)	C36—C27—H27A	108.4 (4)
C15—N4—H4	116.0 (3)	C36—C27—H27B	108.4 (4)
C22—C3—H3A	109.5 (11)	H27A—C27—H27B	107.4 (4)
C22—C3—H3B	109.4 (11)	C6—C33—H33A	109.5 (4)
C22—C3—H3C	109.4 (11)	C6—C33—H33B	109.5 (4)
H3A—C3—H3B	110 (3)	C20—C33—H33A	109.5 (4)
H3A—C3—H3C	110 (2)	C20—C33—H33B	109.4 (3)
H3B—C3—H3C	110 (3)	H33A—C33—H33B	108.0 (5)
C32—C4—H4A	119.2 (5)	C2—C34—H34A	109.7 (4)
C35—C4—H4A	119.0 (5)	C2—C34—H34B	109.6 (4)
C26—C10—H10A	109 (2)	C23—C34—H34A	109.6 (4)
C26—C10—H10B	109.5 (18)	C23—C34—H34B	109.6 (4)
C26—C10—H10C	110 (2)	H34A—C34—H34B	108.2 (5)
H10A—C10—H10B	109 (3)	C7—C36—H36A	109.1 (5)
H10A—C10—H10C	109 (3)	C7—C36—H36B	109.1 (4)
H10B—C10—H10C	110 (3)	C27—C36—H36A	109.0 (4)
C16—C17—H17	119.4 (3)	C27—C36—H36B	109.0 (4)
C22—C17—H17	119.3 (4)	H36A—C36—H36B	107.9 (5)
C12—C18—H18	119.5 (4)	C5—C37—H37A	109.4 (5)
C30—C18—H18	119.4 (4)	C5—C37—H37B	109.4 (6)
C15—C19—H19	119.1 (4)	C24—C37—H37A	109.5 (5)
C26—C19—H19	119.1 (4)	C24—C37—H37B	109.4 (4)
C14—C21—H21	119.4 (4)	H37A—C37—H37B	107.9 (6)
C29—C21—H21	119.4 (4)	C2—C38—H38A	109.4 (19)
C16—C25—H25	120.5 (3)	C2—C38—H38B	109.4 (13)
C31—C25—H25	120.6 (3)	C2—C38—H38C	109.5 (9)
C22—C28—H28	119.6 (4)	H38A—C38—H38B	110 (3)
C31—C28—H28	119.6 (4)	H38A—C38—H38C	110 (3)
C21—C29—H29	120.3 (4)	H38B—C38—H38C	109 (3)

### Tetrabutylammonium bromide–1,1'-(1,2-phenylene)bis(3-m-tolylurea) (1/1) (II) Hydrogen-bond geometry (Å, º)

**Table:**

*D*—H···*A*	*D*—H	H···*A*	*D*···*A*	*D*—H···*A*
N1—H1···Br1	0.86 (1)	2.75 (1)	3.557 (2)	157 (1)
N2—H2···Br1	0.86 (1)	2.51 (1)	3.359 (2)	168 (1)
N3—H3···Br1^i^	0.86 (1)	2.76 (1)	3.420 (2)	135 (1)
N4—H4···Br1^i^	0.86 (1)	2.63 (1)	3.417 (2)	152 (1)
C24—H24*a*···O2	0.97 (1)	2.38 (1)	3.312 (4)	162 (1)
C24^i^—H24*a*^i^···O2^i^	0.97 (1)	2.38 (1)	3.312 (4)	162 (1)
C27^ii^—H27*a*^ii^···Br1^i^	0.97 (1)	3.10 (1)	4.003 (3)	156 (1)

Symmetry codes: (i) −*x*+1, −*y*+1, −*z*+1; (ii) *x*, −*y*+3/2, *z*+1/2.
